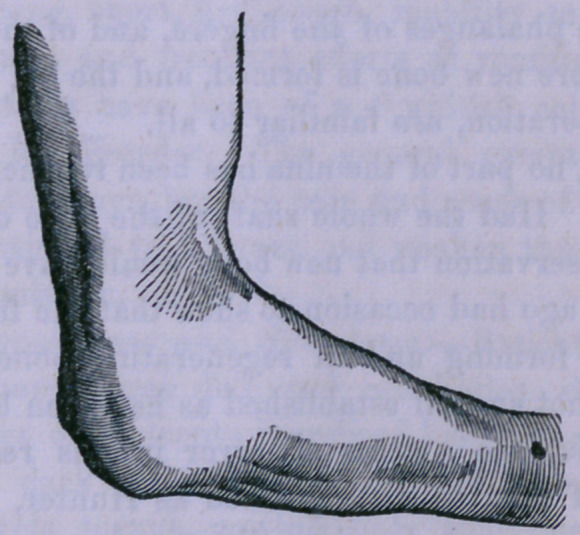# Resection of Two and a Half Inches of the Os Humeri, the Same Extent of the Upper End of the Radius, and Nearly All the Ulna

**Published:** 1858-09

**Authors:** Daniel Brainard

**Affiliations:** Prof. of Surgery in the Medical College of Chicago, etc., etc.


ARTICLE II.

### RESECTION OF TWO AND A HALF INCHES OF THE LOWER END OF THE OS HUMERI, THE SAME EXTENT OF THE UPPER END OF THE RADIUS, AND NEARLY ALL THE ULNA. RECOVERY, WITH A USEFUL MEMBER.

BY DANIEL BRAINARD, M.D.,
Prof. of Surgery in the Medical College of Chicago, etc., etc.

Owen Anderson, aged twenty-six years, Norwegian, a strong, muscular man, consulted me, May 25th, 1857, for a scrofulous disease of the right elbow joint.

*Present State.*—The arm is wasted, and fixed in nearly a straight position; the elbow is greatly enlarged, tender to the touch, and incapable of being moved in any degree, the attempt causing great suffering; the integument about the joint is stretched and shining; the veins are enlarged and filled with dark blood. There are two fistulous openings on the ulnar side of the arm, both communicating with the joint, from which a sero-purulent fluid is constantly discharged. The patient is unable to supinate or pronate the hand. Flexion and extension of the wrist and fingers can be performed only in an imperfect manner, The ulna is enlarged throughout nearly its whole length.

*History.*—The patient states that two years ago he sprained his right elbow joint by having it caught in the machinery of a saw mill. A good deal of swelling and pain followed the injury. These gradually subsided under the application of evaporating lotions, fomentation, etc., so that the patient was able to return to his employment at the end of three weeks; the joint, however, remained tender and painful, especially at night, for two years, the patient in the meantime following his usual occupation, that of a sawyer. At the end of this time, the joint became suddenly very painful and much swollen, so that he was compelled to leave his work and confine himself to his room. Suppuration soon commenced about the joint, and openings were formed; there was loss of appetite and chills. Very little treatment was used during this time, and the patient continued to get worse, the joint all the time enlarging, and being more or less painful up to the present time.

*Operation, May 26th.*—The patient having been placed fully under the influence of chloroform, a transverse incision was carried across the back of the articulation, immediately above the olecranon, from the ulnar nerve to the external condyle, and two longitudinal incisions through the extremities of the transverse, so that the incision altogether had the form of the letter H. The flaps thus formed having been dissected freely back, so as to expose the joint, the olecranon was first removed with the saw; then the internal lateral ligament was divided, and the ulnar nerve pushed over the internal condyle, and two inches of the head of the humerus was removed with the saw; and, lastly, the ends of the radius and ulna were also removed to the extent of two and a half inches. No vessels required ligation. The incisions were closed with the interrupted suture, and water dressings applied, and the arm placed in a flexed position in a trough-like tin splint; one quarter of a grain of morphia was administered, and the patient placed in bed.

Everything went on favorably, the arm being dressed at intervals of two days, with pads of dry lint and a figure of eight bandage, as at the time of the operation, the stitches being removed as they became loose by ulceration, and strips of adhesive plaster substituted till union had become perfect.

On the 28th of June, little more than four weeks after the operation, the elbow was entirely healed. At that time, the arm could be bent and extended nearly to the full extent, and passive pronation and supination were almost perfect.

*July 25th.* Patient called at the office this morning; is able to bend and extend the forearm two-thirds of the limits by voluntary motion, and had very considerable power of supination and pronation. The arm is still gaining strength, and within the last few days he has been able to carry a pail of water in that hand. There still remained, however, a fistulous opening at the point where the ulna had been divided, and the enlargement of this bone was so great as to determine me to remove it.

Accordingly, August 12th, the operation was performed. An incision having been carried along its superficial surface to within three inches of the wrist, and down to the bone, a blunt instrument was insinuated between the bone and periosteum, and pressed backward and forward so as to separate their attachments. This was very readily done, and the ulna sawn across three inches above the wrist, where it was quite sound. This wound healed readily, and, Aug. 24th, there was only one small opening.

*July 25th*, 1858.—This patient paid me a visit. The wound has been perfectly healed for many months, and his health good. The forearm seems shortened to about half its former length. The upper end of the radius rests across the lower end of the humerus. Flexion and extension, to the extent of fifty degrees, are readily performed; rotation, and even lateral movements, to some extent, are also effected. The muscles of the member are well developed, and the patient has been receiving fair wages as a laboring man, and uses the member for feeding and dressing himself very readily.

The accompanying figure correctly represents the appearance of the member at the present time.

*Remarks.*—Since resection of the elbow joint was first proposed by Park in 1780, the operation has been performed so often as to have ceased to attract peculiar attention.

Cases are on record by hundreds, and notwithstanding occasional unfavorable results, the safety and utility of the operation are at the present time well established.

The great majority of these operations, however, only embraced the articular surfaces and extremities of a part or of all the bones concerned in the elbow joint. It is but recently that surgeons have thought of removing the whole or the greater part of one of the bones of the forearm. Professor Carnochan has reported a case of removal of the entire ulna, and another of the radius, in both of which a useful member is stated to have been preserved. But from one case which has fallen under my observation, where the ulna had been removed entire, it might be inferred that amputation of the member would have been preferable.

Had I proposed in the first instance the removal of as much bone as was eventually taken out in this case, I should have advised amputation. The result, however, is a member not only useful but invaluable.

There is one point of interest in a physiological point of view, which must not be overlooked. It is the separation of the bone from the periosteum, in such a manner as to leave this membrane behind almost entire. This was done under the belief that the bone might be to some extent regenerated. The examples of necrosis of the phalanges of the fingers, and of the shaft of the long bones where new bone is formed, and the old eliminated or removed by operation, are familiar to all.

In this case, no part of the ulna has been regenerated. What is the reason? Had the whole shaft of the bone died, we know from ample observation that new bone would have been formed.

I have long ago had occasion to show that the function of the periosteum in forming and in regenerating bone is less considerable and not so well established as has been believed.*

I pointed out the sources of error in this respect, in the experiments of men so distinguished as Hunter, Duhamel and Fleurens. At present, I only call attention to the fact that isolating a long bone from its periosteum and removing it, leaving the periosteum behind entire (an operation which can, in some cases of disease of the bone, be readily performed), does not necessarily lead to a regeneration of bone.

---

*Essay on the Treatment of Ununited Fractures and Certain Deformities of the Osseous System. Paris, 1853.

## Figures and Tables

**Figure f1:**